# Regulation and use of the extracellular matrix by Trypanosoma cruzi during early infection

**DOI:** 10.3389/fimmu.2012.00337

**Published:** 2012-11-06

**Authors:** Pius N. Nde, Maria F. Lima, Candice A. Johnson, Siddharth Pratap, Fernando Villalta

**Affiliations:** Department of Microbiology and Immunology, School of Medicine, Meharry Medical CollegeNashville, TN, USA

**Keywords:** laminin γ-1, thromospondin-1, calreticulin, gp83, ECM interactome, cellular infection, *Trypanosoma cruzi*, systems biology

## Abstract

Chagas disease, which was once thought to be confined to endemic regions of Latin America, has now gone global becoming a new worldwide challenge. For more than a century since its discovery, it has remained neglected with no effective drugs or vaccines. The mechanisms by which *Trypanosoma cruzi* regulates and uses the extracellular matrix (ECM) to invade cells and cause disease are just beginning to be understood. Here we critically review and discuss the regulation of the ECM interactome by *T. cruzi*, the use of the ECM by *T. cruzi* and analyze the molecular ECM/*T. cruzi* interphase during the early process of infection. It has been shown that invasive trypomastigote forms of *T. cruzi* use and modulate components of the ECM during the initial process of infection. Infective trypomastigotes up-regulate the expression of laminin γ-1 (LAMC1) and thrombospondin (THBS1) to facilitate the recruitment of trypomastigotes to enhance cellular infection. Silencing the expression of LAMC1 and THBS1 by stable RNAi dramatically reduces trypanosome infection. *T. cruzi* gp83, a ligand that mediates the attachment of trypanosomes to cells to initiate infection, up-regulates LAMC1 expression to enhance cellular infection. Infective trypomastigotes use Tc85 to interact with laminin, p45 mucin to interact with LAMC1 through galectin-3 (LGALS3), a human lectin, and calreticulin (TcCRT) to interact with TSB1 to enhance cellular infection. Silencing the expression of LGALS3 also reduces cellular infection. Despite the role of the ECM in *T. cruzi* infection, almost nothing is known about the ECM interactome networks operating in the process of *T. cruzi* infection and its ligands. Here, we present the first elucidation of the human ECM interactome network regulated by *T. cruzi* and its gp83 ligand that facilitates cellular infection. The elucidation of the human ECM interactome regulated by *T. cruzi* and the dissection of the molecular ECM/*T. cruzi* interphase using systems biology approaches are not only critically important for the understanding of the molecular pathogenesis of *T. cruzi* infection but also for developing novel approaches of intervention in Chagas disease.

## LAMC1 AND THBS1 ARE REQUIRED FOR *T. cruzi* INFECTION

As part of our efforts to identify the molecular signatures induced by *Trypanosoma cruzi *in mammalian cells during the early infection process, we analyzed the kinetics of the extracellular matrix (ECM) human transcriptome response. The result of this analysis showed that the only ECM proteins, LAMC1 and THBS1, were important in the early infection process. Accordingly, silencing the expression of LAMC1 and THBS1 by stable RNAi significantly reduced *T. cruzi* infection in cells (Nde et al., 2006; Simmons et al., 2006). The cells in which stable LAMC1 RNAi or THBS1 RNAi were performed showed significant reduction in the protein expression level of LAMC1 or THBS1 compared to cells stably transfected with vector alone or scrambled LAMC1 or THBS1 and the kinetics of *T. cruzi* infection were also dramatically reduced (Nde et al., 2006; Simmons et al., 2006). These studies showed that *T. cruzi* requires LAMC1 and THBS1 for early infection and indicated that host LAMC1 and THBS1 play critical roles in the early process of *T. cruzi* infection (Nde et al., 2006; Simmons et al., 2006). The important roles played by LAMC1 and THBS1 encouraged our group to dissect their molecular role in *T. cruzi* infection by looking at the ECM/*T. cruzi* interphase and elucidating the gene-networks and interactomes triggered by *T. cruzi* and its surface molecules involved in the early infection process (Cardenas et al., 2010; Nde et al., 2010). This will be critically reviewed in the next sections.

## *T. cruzi* SURFACE GP83 UP-REGULATES LAMC1 TO RECRUIT PARASITES AT THE ECM TO ENHANCE INFECTION

Gp83 is a ligand expressed in all *T. cruzi* strains and employed by the parasite to attach and enter macrophages as well as non-phagocytic cells (Lima and Villalta, 1988; Villalta et al., 1998, 1999, 2001, 2008). Notably, it is expressed only in invasive trypomastigotes (Villalta et al., 1992) and is more expressed in highly infective trypomastigote clones (Lima and Villalta, 1989). Monovalent Fab fragments of the gp83-specific monoclonal antibody 4A4 inhibit gp83 binding to myoblasts, fibroblasts, and macrophages, block trypanosomes from attaching to and entering these cells, and neutralize *T. cruzi* infection *in vivo* (Villalta et al., 2001). Trypomastigotes release gp83 via parasite glycosylphosphatidylinositol–phospholipase C (PLC) cleavage to activate the host MAPK pathway and PKC in order to promote parasite infection (Villalta et al., 1998, 1999; Nde et al., 2006).

Exposure of gp83 ligand to human cells increases the level of LAMC1 transcripts and its expression in mammalian cells, leading to an increase in cellular infection by *T. cruzi* (Nde et al., 2006). This increase in cellular infection was seen as over-attachment and entry of trypomastigotes into human cells over-expressing LAMC1, under the influence of gp83 ligand, resulting in high parasite multiplication at 72 h. These observations together with the fact that knocking down the expression of LAMC1 by RNAi dramatically reduces *T. cruzi* attachment, entry and multiplication within cells strongly support the hypothesis that host LAMC1, which is regulated by the parasite gp83, plays a crucial role in the early process of cellular infection.

The regulation of infection by the gp83 ligand represents a parasite escape mechanism in which invasive trypomastigotes release gp83 to efficiently gain entry into human coronary artery smooth muscle (HCASM) cells by manipulating LAMC1, which is the most abundant isoform of laminin in humans (Sasaki et al., 2004).

*Trypanosoma cruzi* must navigate through the basal lamina, which contains LAMC1, and surrounds individual muscle cells such as HCASM cells before infecting these cells. The fact that a *T. cruzi* trypomastigote ligand increases LAMC1 transcript levels in HCASM cells, correlates well with the finding that laminin is deposited in the hearts of patients infected with Chagas’ disease (Milei et al., 1993). This suggests that the regulation of LAMC1 in heart cells by gp83 may be involved in heart pathology.

Since the *T. cruzi* gp83 ligand remodels the ECM by up-regulating the expression of LAMC1, together with the report that *T. cruzi* presents laminin receptors on its surface (Giordano et al., 1999), indicates that the parasite exploits LAMC1 to navigate through the ECM, recruit trypanosomes and facilitate infection. Thus, the *T. cruzi* gp83 ligand is a virulence factor that modifies LAMC1 expression in the ECM and contributes to the pathogenesis of *T. cruzi* infection in human heart cells.

## *T. cruzi* MODULATES THE LAMC1 SUB-NETWORK

Recently, the first LAMC1 sub-network modulated by invasive trypomastigotes during the early process of infection was elucidated (Nde et al., 2010). To populate and build the interaction network, the Michigan Molecular Interactions (MiMI) cytoscape plugin (version 3.2) was used. MiMI retrieves molecular interactions from MiMI database and displays the interaction network with cytoscape. MiMI gathers and merges data from well-known protein interaction databases including BIND, DIP, HPRD, RefSeq, SwissProt, IPI, and CCSB-HI1. The plugin also integrates with other NCBI tools for literature information, document summarization, and pathway matching (Gao et al., 2009). The RT-PCR verified transcripts were used as the initial population nodes. MiMI was used to query for the initial nodes and their respective nearest neighbors to one degree. The networks were then merged for interconnections and the interactome was visualized in cytoscape (version 2.6.1) using the yFiles: organic layout format (Cline et al., 2007).

The first elucidated LAMC1 sub-network interactome mobilized in cells by *T.* cruzi involves the following genes CCDC53, NID1, NID2, SNAPIN, LAMA1, SV2A, CD44, LAMA5, ATF71P, ITGB4, LAMB1, LAMB2, SV2C, SV2B, ITGA6, and BCL6 (Nde et al., 2010). Our group is fully investigating the roles of the members of this sub-network in the infection process. Accordingly, the ITGA6 gene that encodes integrin alpha-6 that combines with beta 4 as a laminin receptor, or with beta 1 in the integrin VLA-6 participates in cell adhesion as well as cell-surface mediated signaling. The integrin, beta 4 (ITGB4) gene that encodes the integrin beta 4 subunit, a receptor for the laminins, tends to associate with alpha 6 subunit and appears to be involved in cell invasion. Entactin, also known as nidogen-1 (NID1) is a component of the basement membrane that connects the networks formed by collagens and laminins to each other and plays a role in cell interactions with the ECM. Furthermore, LAMA1 (Laminin, alpha 1) interacts with Fibulin-2 (FBLN2), an ECM protein that binds various extracellular ligands and calcium and appears to be involved in the infection process. Interestingly, BCL6 is a central hub of the interaction LAMC1 sub-network, which is also a common central hub to five of the seven sub-networks reported by one degree of connection to the initial seed nodes (Nde et al., 2010).

The elucidation of the LAMC1 sub-network interactome mobilized by *T. cruzi* is critically important to understand the molecular pathogenesis of *T. cruzi *infection derived from the ECM/*T. cruzi* gp83 ligand interplay and for the development of approaches for intervention. We expect that continuing to explore systems biology in the early process of *T. cruzi* infection will rapidly bring novel approaches for the treatment and management of Chagas disease.

## THBS1 INTERACTS WITH *T. cruzi* SURFACE CALRETICULIN TO ENHANCE CELLULAR INFECTION

Thrombospondins have been described as “matricellular proteins” because they play a role in regulating cellular responses and ECM remodeling in the pericellular microenvironment but they are non-essential components of the mature matrix fibrils (Bornstein, 2002). The role of THBS1 *in vitro* and *in vivo* is complex and context specific, because it interacts with a wide array of cellular proteins. THBS1 is a large homotrimeric glycoprotein containing several domains that can bind to cell surface receptors and extracellular molecules (Chen et al., 2000) such as the N-terminal heparin-binding domain (NTSP), procollagen region, type 1, 2, and 3 repeats and a C-terminal domain (Elzie and Murphy-Ullrich, 2004). The molecule also contains highly conserved epidermal growth factor (EGF) repeats, type 3 repeats and a C-terminal domain, which includes the signature domain (Carlson et al., 2005) that can interact with integrins and CD47 (Lawler and Hynes, 1989; Gao and Frazier, 1994). The C-terminal domain of the thrombospondin family is highly conserved compared to the N-terminal domain, which is different for each thrombospondin isoform.

Calreticulin (CRT) is a major intracellular well-conserved calcium-binding chaperone, which was identified in skeletal muscle (Michalak et al., 1992) and is present in the cells of all higher organisms except erythrocytes (Michalak et al., 1999; Johnson et al., 2001a,b). Numerous reports have implicated CRT in several cellular functions and the molecule has significant non-endoplasmic reticulum functions in normal physiology and human disease status (Gold et al., 2010).

Calreticulin has also been described in some parasite species such as *Schistosoma mansoni*, *Onchocerca volvulus, Necator americanus, Leishmania donovani*, and *Plasmodium falciparum.* However, the role that this protein might play in parasite interactions with the host immediate microenvironment remains unknown (Nakhasi et al., 1998; Ferreira et al., 2004, 2005).

Previous studies from our group showed that invasive *T. cruzi* trypomastigotes up-regulate the expression of THBS1 in HCASM cells (Simmons et al., 2006). Furthermore, knockdown of THBS1 rendered mammalian cells less susceptible to cellular infection by *T. cruzi* indicating that THBS1 also plays an important role in the process of cellular infection by *T. cruzi*. However, the mechanisms by which THBS1 is up-regulated by the parasite to modulate cellular infection are not completely known. The elucidation of *T. cruzi* surface molecules that interact with THBS1 to enhance cellular infection will advance our understanding of the molecular pathogenesis of *T. cruzi* infection. We anticipated that NTSP, which is specific to this isoform of thrombospondin, would be essential in the interaction with the parasite because it is different for all thrombospondin isoforms compared to the conserved C-terminal domain (Carlson et al., 2005).

In *T. cruzi*, it has been suggested that the parasite surface CRT (TcCRT; Ferreira et al., 2004; Aguilar et al., 2005) could play a role in enabling the pathogen to evade the host immune response by interacting with the C1q component of complement (Aguilar et al., 2005). The mechanism by which the parasite CRT interacts with host proteins to enhance the process of cellular invasion remains unknown. We recently hypothesized that *T. cruzi* uses its surface TcCRT to exploit matricellular proteins regulated by the parasite to enhance cellular infection.

**FIGURE 1:**
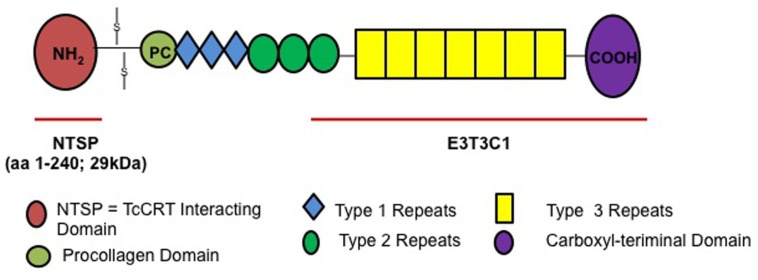
**THBS1 structure and NTSP domain that interacts with TcCRT to enhance *T. cruzi* cellular infection**.

We recently reported that *T. cruzi* up-regulates the expression of THBS1 to enhance the process of cellular invasion. Very recently our group characterized a novel THBS1 interaction with *T. cruzi* that enhances cellular infection (Johnson et al., 2012). We showed that labeled THBS1 interacts specifically with the surface of *T. cruzi* trypomastigotes. In **Figure 1** we show the full-length THBS1 and the NTSP domain that interact with TcCRT. Pre-exposure of recombinant NTSP or THBS1 to *T. cruzi* significantly enhanced cellular infection of wild-type mouse embryo fibroblasts (MEF) compared to the C-terminal domain of THBS1, E3T3C1. In addition, blocking TcCRT with antibodies significantly inhibited the enhancement of cellular infection mediated by the TcCRT–THBS1 interaction. Taken together, these findings indicate that THBS1 interacts with TcCRT on the surface of *T. cruzi* through the NTSP domain and that this interaction enhances cellular infection. Thus, surface TcCRT is another virulence factor that enhances the pathogenesis of *T. cruzi* infection through THBS1, which is up-regulated by the parasite.

The observation that THBS1 interacts with the parasite membrane TcCRT is also in agreement with the recent discovery that CRT, which was previously thought to be exclusively intracellular, is also expressed on the surface of the parasite (Arosa et al., 1999; Xiao et al., 1999; Yan et al., 2010). The identification of CRT homologs in other parasites such as *Onchocerca*, *Schistosoma*, and *Leishmania* (Michalak et al., 1992; Nash et al., 1994; Joshi et al., 1996) suggests that the protein functions as an intracellular chaperone but its role in the process of infection of those parasites remains unknown.

Our group showed that surface TcCRT is a virulence factor that interacts with host THBS1 to enhance cellular infection by *T. cruzi* (Johnson et al., 2012). In order to explore the significance of host THBS1 and TcCRT in cellular infection by *T. cruzi*, our group used THBS1 KO MEF and WT MEF in infection assays. We observed that host THBS1 and parasite surface TcCRT are important for MEF cellular infection by *T. cruzi*. The significance of surface TcCRT in enhancing cellular infection by *T. cruzi* was supported by the fact that specific antibodies to TcCRT significantly reduced cellular infection. The identification of TcCRT as a virulence factor expressed on the surface of the parasite can be exploited to provide new insights into the molecular pathogenesis of *T. cruzi* infection. TcCRT expression on the parasite surface may modulate the vertebrate complement system as an immune escape mechanism (Ferreira et al., 2005). Taken together, these findings indicate that THBS1 interacts with TcCRT on the surface of *T. cruzi* through the NTSP domain and that this interaction enhances cellular infection by *T. cruzi*.

## *T. cruzi* MODULATES THE THBS1 SUB-NETWORK

The matricellular THBS1 glycoprotein inhibits angiogenesis and modulates endothelial cell adhesion, motility, and growth. THBS1 interacts with several cell adhesion receptors, including CD36, integrins, and integrin-associated proteins and inhibits matrix metalloproteinase enzymes thereby remodeling the cellular microenvironment (Sid et al., 2004). We have shown that *T. cruzi* up-regulates the expression of THBS1 to facilitate the invasion process (Simmons et al., 2006). Knockdown of THBS1 by RNAi significantly inhibited cellular invasion by *T. cruzi. *The increased level of *THBS1* expression significantly modulates the interactome cross-talk between the cells, as we found the largest protein–protein interaction (PPI) sub-network occurred in the THBS1 seed network, with 51 nodes interconnected with 151 edges (Nde et al., 2010). This network topology will potentially favor parasite multiplication and survival. During this cross-talk, higher levels of THBS1 activate TGF-β (Ribeiro et al., 1999), a major pro-fibrotic cytokine causing modification of the ECM milieu. THBS1 then interacts with several host proteins including the COL7A1 gene product, which then interacts with other proteins from the laminin family of genes to make the ECM conducive to parasite mobility and cellular invasion. The modulation of THBS1 gene networking profile is highly critical for trypanosome mobility and cellular invasion. Other parasites (including the human pathogens *Plasmodium*, *Toxoplasma*, and *Cryptosporidium*), which do not regulate human THBS1, use a transmembrane thrombospondin-related anonymous protein (TRAP) for gliding motility and invasion of vertebrate host cells (Kappe et al., 1999; Sibley, 2004; Morahan et al., 2009). TRAP proteins produced by the parasite can also activate TGF-β, an anti-inflammatory cytokine that counteracts the effects of inflammatory cytokines like IL-12, INF-γ, and TNF-α thereby facilitating parasite survival. TSP1, also binds to matrix metalloproteinase-2(MMP-2) and appears to be involved in the infection process.

Modulation of THBS1 expression in HCASM cells by *T. cruzi* is essential because THBS1 blocks the cytoprotective activity of nitric oxide (NO) by antagonizing the NO/cGMP signaling pathway thereby negatively regulating vascular tone, vascular smooth muscle cells adhesion, chemotaxis, and proliferation (Isenberg et al., 2008). Increased THBS1 transcripts in parasitized HCASM cells may suggest that THBS1 also contributes in part to the pathology caused by *T. cruzi*. THBS1 is required for the infection process of *T. cruzi* as evidenced by RNAi of that specific isoform. The up-regulation of host THBS1 expression by *T. cruzi* to facilitate the infection of human cells represents an additional mechanism that contributes to the pathogenesis of *T. cruzi* infection.

The first elucidated THBS1 sub-network interactome mobilized in cells by *T. cruzi* involves the following genes: TSC2, TNFRSF1A, COL1A1, COL2A1, COL3A1, COL5A1, COL7A1, SDC1, SDC2, SDC3, SCD4, SPARC, LTBP1, HRG, ITGB1, ITGB3, ITGA3, ITGA4, ITGAV, FGA, SPP1, F2, CD47, MMP2, MMP9, CALR, TFPI, ZNF8, LRP1, LRP5, TP53, CTSG, PDGFB, ELA2, CORO1A, LAMB3, CFH, PDEA, JAG1, DCN, TGFB1, CD36, FN1, PLG, TNFAIP6, SCARB2, DHFR, and KNG1 (Nde et al., 2010). The roles of the members of the THBS1 sub-network in the infection process are also being investigated fully by our group as indicated above in this section. The new advances in the area will facilitate the identification of the molecular signature induced by *T. cruzi* in cells via the TcCRT–THBS1 interphase as well as the development of small molecule inhibitors to interrupt the critical initial steps of *T. cruzi* infection.

## *T. cruzi* USES LAMININ AND GALECTIN-3 TO PROMOTE CELLULAR INFECTION

The ECM, human lectins, and parasite mucins have been shown to play an important role in the early process of *T. cruzi* infection. Accordingly, human galectin-3 binds to a trypomastigote surface mucin (Moody et al., 2000; Turner et al., 2002) and to HCASM cells in a lectin-like manner (Kleshchenko et al., 2004) to significantly increase the adhesion of trypomastigotes to these cells. Silencing galectin-3 expression in cells by antisense approach significantly reduces trypomastigote cell adhesion. Galectin-3 molecules interact with a *T. cruzi* 45 kDA mucin surface protein on one hand and with laminin on the other, via their carbohydrate recognition domains and are joined together through the R-domains (Moody et al., 2000; Kleshchenko et al., 2004). In this way, galectin-3 binds to laminin and trypomastigotes to recruit them to the ECM thus facilitating initial infection. Thus, galectin-3 provides a bridge between parasites and laminin in host cell thereby enhancing infection. Prior to cellular infection the infective trypomastigotes bombard host cells with PLC-cleaved gp83 to activate the ERK1/2 pathway to up regulate laminin primed cells to enhanced infection via the laminin-45 mucin-galectin-3 pathway (Moody et al., 2000; Kleshchenko et al., 2004). These studies pointed out a novel *T. cruzi*–host cell interaction mediated by gp83-laminin-45 mucin- galectin-3 that recruits significant number of parasites at the ECM to facilitate cellular infection. Other *T. cruzi* surface antigens bind to laminin (Giordano et al., 1999) and fibronectin (Ouaissi et al., 1986) and have been postulated to participate in the infection process, however these interactions have not been studied using systems biology approaches. Overall, we can conclude that the parasite modulates some ECM components and interacts with them to facilitate infection by exploiting these molecules and human lectins to recruit parasites in the early process of infection.

Galectin-3 binds to the surface of HCASM cells in a granular manner, distributed around the cellular membrane, polarized, and more pronounced at the cellular ends (Kleshchenko et al., 2004). The receptors for human galectin-3 are distributed in patches on the cell surface and are more abundant at the cellular terminal regions. The binding of galectin-3 to trypomastigotes is also seen as granular, restricted to some areas of the trypanosome membrane, and polarized (Kleshchenko et al., 2004).

Galectin-3 is also implicated in the association of *T. cruzi* trypomastigotes with laminin (Moody et al., 2000). Binding of trypomastigotes to laminin is enhanced by galectin-3 and this enhanced binding of trypanosomes is inhibited by lactose. Co-immunoprecipitations indicate that galectin-3 binds to the 45 kDa trypomastigote surface protein and this binding is also inhibited by lactose (Moody et al., 2000). The monoclonal antibody B5 that recognizes the trypomastigote 45 kDa surface mucin blocks trypomastigote attachment to heart myoblasts (Moody et al., 2000; Turner et al., 2002). A working model proposes that galectin-3, released by human cells, forms bridges between *T. cruzi* and laminin. Since nearly all the tissues which *T. cruzi* infects are surrounded by basement membranes of which laminin is a major constituent, its ability to effectively interact with laminin is critically important for passage through the membrane barrier. These studies suggest that this is a trypanosome trapping mechanism, which enables the organisms to accumulate in the basement membrane prior to invasion of heart myoblasts, making galectin-3 a candidate molecule, which enhances the pathogenesis of *T. cruzi *via laminin and a *T. cruzi* surface mucin.

Galectins have long been suspected of modulating cell to ECM interactions in a novel fashion (Liu and Rabinovich, 2005). Data suggest that one mechanism involves the ligation of mammalian cells to ECM proteins, which also interact with galectin-3 such as laminin and elastin (Liu and Rabinovich, 2005). The other mechanism involves the interaction of galectins with the polylactosamine residues of integrins, resulting in the modulation of cellular adhesion to ECM proteins (Rabinovich et al., 2002). Whereas most of these studies were done in mammalian systems, it has been suggested that galectins expressed by *Entamoeba* may be critical in their interactions with host cells (Petri et al., 1990). It is likely that in parasitic organisms, the binding of the organisms to the ECM proteins or cell surface glycoconjugates may be the primary adhesion mechanisms mediated by galectins.

Galectin-3 is also expressed in B cells from *T. cruzi*-infected mice (Acosta-Rodríguez et al., 2004) and is up-regulated by *T. cruzi* infection of mice (Vray et al., 2004). The fact that galectin-3 is secreted by macrophages and by other cells, including HCASM cells, suggests that released galectin-3 modulates infection via laminin. The concentrations of galectin-3 that increase trypanosome adhesion to HCASM cells *in vitro* are similar to the concentrations of galectin-3 present in fluids *in vivo* (Sano et al., 2000). Furthermore, the concentrations of galectin-3 in fluids *in vivo* increase approximately 300-fold during microbial infection (Sato and Hughes, 1994). These observations suggest that the parasite may have adapted to the host and that it takes advantage of a host inflammatory molecule, galectin-3, to bind to host cells via laminin and a *T. cruzi* mucin to enhance infection.

Previous studies have shown that parasite molecules bind to immobilized laminin (Giordano et al., 1999) and that human galectin-3 enhances this interaction (Moody et al., 2000), indicating that the trypanosome interacts with laminin via galectin-3.

## *T. cruzi* SURFACE GP83 REGULATES THE ECM INTERACTOME

**FIGURE 2:**
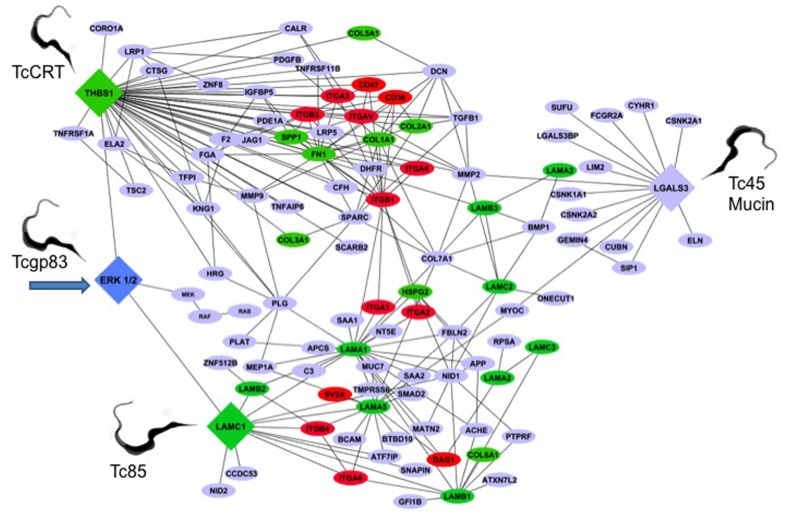
***Trypanosoma cruzi* gp83 regulates the ECM interactome and trypanosome usage of the ECM during early infection to enhance cellular infection.** *T. cruzi* gp83 ligand triggers gp83 receptors in the cell via ERK1/2 to up-regulate LAMC1 which cross-talks with LGALS3 and THBS1 to enhance cellular infection using selected parasite surface molecules such as TcCRT, TC45 mucin, and Tc85. Arrow points to the first signal. The interactome was generated using MiMI Cytoscape plugin (version 3.2). The MiMI interface uses a database which is itself constructed from merged data of well-known protein interaction databases as described in the text of the review. The network was connected by using ERK1/2, Laminin gamma-1 (LAMC1), Thrombospondin-1 (THBS1), and Galectin-3 (LGALS3) as initial seed nodes (large DIAMOND nodes). Node expansion was done to one degree of biological protein–protein interaction partners of the seed nodes using the Michigan Molecular Interaction Database. Proteins mapping to the canonical “ECM-Receptor Interaction Pathway” of KEGG (hsa04512) are highlighted as following: GREEN nodes are ECM ligands (laminins, collagens, fibronectin, heparan sulfate, and thrombospondin-1). RED nodes are ECM receptors: (integrins, cd35, cd47, and SV2A). BLUE nodes are not part of the KEGG ECM–ECM Receptor Pathway, but have established biological interactions with ECM–Receptor interaction nodes. The global interactome was visualized in Cytoscape (version 2.6.3), yielding the ECM interactome involved in early *T. cruzi* infection. Trypomastigotes pointed in the figure are culture trypomastigotes released from infected cells expressing surface Tcgp83, TcCRT, and Tc45 mucin. All of these trypomastigote surface molecules are also expressed in blood trypomastigotes.

The genetic architecture of the early *T. cruzi* infection process of human cells is very limited. To understand this aspect of the infection, we conducted gene transcription microarray analysis followed by gene network construction of the host cell response in primary HCASM cells infected with *T. cruzi* or exposed to *T. cruzi* gp83. Using THBS1, LAMC1, LGALS3, and ERK1/2 as the seed nodes for biological network construction, we built an interactome network of the early *T. cruzi* infection process, which centered on the ECM. ERK1/2 was used as one seed mode, since *T. cruzi *gp83 activates ERK1/2 in cells to up-regulate the LAMC1 expression and infection. After seeding the initial four nodes, the network was expanded to one degree of direct biological interaction, resulting in base interaction networks with the seed node as a center point in each. In order to populate and build our interaction network we used a similar approach to determine the modulation of the LAMC1 sub-network as indicated above. **Figure 2** shows the ECM interactome that is modulated by *T. cruzi* gp83 ligand. The first step in the regulation of the ECM interactome by *T. cruzi* during the initial infection process involves triggering gp83 receptors in the cell via activation of ERK1/2 by PLC-cleaved gp83 which results in the up-regulation of LAMC1 and activation of the LAMC1 sub-network which directly cross-talks with the LGALS3 sub-network to recruit parasites and enhance cellular infection on one side. On the other side, triggering gp83 receptors in the cell via ERK1/2 cross-talk with the THBS1 sub-network, also enhances cellular infection and cross-talk at a distance with LGALS3, which also recruit parasites via Tc45 mucin, and LAMC1 (**Figure 2**). Tc85 interacts with laminin and may mobilize the ECM interactome. It is also possible that *T. cruzi* MMP9 like activity that degrades the ECM may regulate the ECM interactome (Nogueira de Melo et al., 2010). This ECM-focused interactome contains 108 nodes representing protein-coding genes connected by 222 edges representing biological interactions between nodes (**Figure 2**).

Increased THBS1 expression significantly modulates the interactome cross-talk between cells. This change in network topology potentially favors parasite invasion and infection of host cells. As such, THBS1 interacts with several proteins ranging from adhesion receptors (CD36 and CD47) to structural proteins (COL7A1) and zymogens. CD47 is a receptor for the C-terminal domain of THBS1 and this interaction may be important in membrane transport and signal transduction. In addition, CD47 is involved in intracellular calcium increase, which occurs when the cell adheres to the ECM. An increase in cytosolic Ca^++^ in *T. cruzi* trypomastigotes was detected at the single cell level after association of the parasites with myoblasts. Ca^++^ mobilization in host cells was also detected upon contact with trypomastigotes. Confirmatively, pretreatment of the parasites with the Ca^++^ chelators decreased trypomastigote association to myoblasts indicating that calcium mobilization is required for cell invasion (Moreno et al., 1994).

The structural protein COL7A1 is typically found in the basement membrane and also associates with THBS1. Once COL7A1 interacts with THBS1, it makes the ECM conducive to parasite motility and cellular invasion with the help of proteins from the laminin family of genes. Plasminogen (PLG) links LAMC1 and THBS1. PLG (a zymogen) is cleaved into plasmin (a serine protease), and angiostatin (an angiogenesis inhibitor). Plasmin is known to cleave fibronectin, THBS1, and LAMC1, thus taking part in ECM modifications.

*Trypanosoma cruzi* must navigate through the basal lamina, which contains LAMC1. The *T. cruzi* gp83 ligand modifies LAMC1 expression in the ECM and contributes to the pathogenesis of *T. cruzi* infection in human heart cells. The fact that the LAMC1 network is connected to THBS1 through PLG and COL7A1 and matrix metalloproteinase 2 (MMP2) suggests that this network could facilitate parasite mobilization. MMP2 is a type IV collagenase that participates in the rearrangement of the ECM, which could facilitate parasite mobilization. In the LAMC1 sub-network there is an indirect connection (through LAMA1) to dystroglycan (DAG1). DAG1 is a dystrophin-associated glycol-protein responsible for transmembrane linkage between the ECM and host cell cytoskeleton. The extracellular form of DAG1 can bind to merosin alpha-2-laminin in the ECM (Yamada et al., 1994). If *T. cruzi* alters the dystroglycan complex, it could consequently manipulate or weaken the host cell cytoskeleton prior to gaining entry into the cell. This could yet be another mechanism by which *T. cruzi* invades host cells. In reference to the ECM network reviewed, LAMC1 also has a second-degree interaction (one node in between the genes) with LGALS3 through myocilin (MYOC). MYOC is a secreted protein believed to have a role in cytoskeletal function, specifically vesicular transport and ECM conformation (Caballero et al., 2000). Unlike other intracellular pathogens, which avoid contact with host cell lysosomes, *T. cruzi* requires the low-pH environment of lysosomes to initiate egression from the vacuole and delivery to the host cell cytoplasm where replication takes place (Burleigh, 2005). Therefore, the control of vesicular trafficking by *T. cruzi* improves the rate of trypomastigote entry and amastigote replication in the host cell. The fact that LAMC1 is connected to LGALS3 through MYOC in the ECM interactome suggests the importance of LGALS3 in the manipulation of host ECM by *T. cruzi*.

Increased LGALS3 expression in the ECM promotes the adhesion of *T. cruzi* to host cells and subsequent infection (Kleshchenko et al., 2004). In addition, LGALS3 has numerous ECM interacting partners (Dumic et al., 2006), including collagen IV, hensin, laminins, fibronectin, vitronectin, tenascin, and elastin. LGALS3 regulates adhesion of these ECM proteins to a variety of host cells. Matrix metalloproteases, which are more active in *T. cruzi* infected mice, regulate LGAL3 function (Gutierrez et al., 2008). When metalloproteases are activated in the ECM, they can cleave LGALS3 and negatively regulate its function. Consequently, increased activation of MMP1 and MMP9 is associated with ECM destruction and myocarditis in *T. cruzi* infection.

Multiple types of collagen interact with the three central seed nodes, THBS1, LGALS3, and LAMC1. A glimpse of the importance of collagen in early *T. cruzi* infection was reported (Velge et al., 1988).

## SOME POTENTIAL IMPLICATIONS OF THE ECM IN INFECTION

Expression profiling microarray studies of *T. cruzi* infected cells have shown that some ECM genes are regulated (Goldenberg et al., 2009; Mukherjee et al., 2003), however those genes were not confirmed by RT-PCR, nor their function in the process of *T. cruzi* infection was determined. Several reports suggest that some parasite proteases may degrade the ECM. The possible role of trypanosomatid surface proteases in parasite survival and infection has been discussed (Marino et al., 2003; Yao, 2010). It was suggested that the gp85/trans-sialidase interacts with the ECM components (Alves and Colli, 2008) and speculated that the prolyl oligopeptidase Tc 80 may be involved in the infection possibly by acting on ECM components (Grellier et al., 2001). Interestingly, it was found that in a *T. cruzi* infected mouse embryo hepatocyte cell line, there was a reduction of MMP9 (Nogueira de Melo et al., 2004), as well as some matrix components during the late phase of infection (60th to 90th day; Magalhaes-Santos et al., 2002). Pinho et al. (2002) reported that uncharacterized parasite antigens bind to non-infected cells and that ECM components were recognized by antibodies, however the molecular characterization of antigens and the specificity of these possible interactions were not considered. Furthermore, Santana et al. (1997) showed that a Tc 80 proteinase hydrolyzes collagen type in rat mesentery. It is possible that that ECM derived from *T. cruzi* infected endothelial cells directs phenotypic expression (Morris et al., 1990).

It has been suggested that there are fibrotic implications during *T. cruzi* infection involving the ECM. Accordingly, Calvet et al. (2009) suggested that TGF-β and TNF-α stimulate fibronectin expression in uninfected cells of *T. cruzi*-infected cultures, whereas cells harboring the parasites display low or no fibronectin fibrils. It was observed that there are sequential changes of the connective matrix components of the myocardium (fibronectin and laminin) during fibrosis in infected mice (Andrade et al., 1989). The fact that *T. cruzi*-mediated down-regulation of CTGF expression requires *de novo* host cell protein synthesis and that *T. cruzi* interferes with the host fibrogenic response suggest that this complex process requires input from multiple host cell signaling pathways (Unnikrishnan and Burleigh, 2004). Although it has been demonstrated that cardiomyocytes are able to synthesize cytokines upon *T. cruzi* infection, Calvet et al. (2004) suggest that matrix remodeling is dependent on cytokines secreted by inflammatory cells recruited in immune response. These reports suggest that *T. cruzi* modulates the ECM during the infection process with potential implications in the pathology of the disease.

## CONCLUDING REMARKS

Despite an appreciation of the involvement of the ECM in *T. cruzi* infection, the difficulty in delineating regulatory networks in the process of cellular infection and disease, has until now prevented a comprehensive assessment of *T. cruzi* infection at the molecular level. The use of systems biology approaches to elucidate the ECM interactome network regulated by *T. cruzi* and its gp83 ligand that mediate trypanosome attachment and entry are critically important to understand the molecular pathogenesis of *T. cruzi* infection and to design novel approaches for intervention and disease management. There is an expectation that systems biology approaches applied to understand *T. cruzi* infection would bring new innovative strategies for the treatment and control of Chagas disease. So far the use of systems biology to understand the role of the ECM in *T. cruzi *infection has significantly enhanced the understanding of *T. cruzi*–host cell interaction and pathogenesis of *T. cruzi* infection. Accordingly, here we elucidate the first ECM interactome triggered by the *T. cruzi* gp83 ligand during the first step of cellular infection to recruit trypanosomes at the ECM to enhance cellular infection. In the shot gun used by *T. cruzi* to regulate the ECM interactome to gain cellular entry and evade the host reported here, *T. cruzi* gp83 triggers gp83 receptors in the cell via ERK1/2 to up-regulate LAMC1 which cross-talks with both LGALS3 and THBS1 to enhance cellular infection using selected parasite surface molecules such as TcCRT and TC45 mucin. Based on this new information, current efforts are being pursued by our group to elucidate the molecular signature induced by *T. cruzi* in cells. Since *T. cruzi*–host cell interaction is of a complex nature, the elucidation of the global interactome and sub-interactome networks will help understand the complex *T. cruzi*–host interphase that mediate parasite attachment and entry, and develop new means for the treatment of the highly neglected and complex Chagas disease, which has no current effective drugs or vaccines.

## Conflict of Interest Statement

The authors declare that the research was conducted in the absence of any commercial or financial relationships that could be construed as a potential conflict of interest.

## Abbreviations

4A4: gp83 monoclonal antibody that neutralizes *T. cruzi* infection
ACHE: acetylcholinesterase (Yt blood group)
APCS: amyloid P component, serum
APP: amyloid beta (A4) precursor protein (peptidase nexin-II, Alzheimer disease)
ATF7IP: activating transcription factor 7 interacting protein
ATXN7L2: ataxin 7-like 2
B5: *T. cruzi* p45 mucin monoclonal antibody
BCAM: basal cell adhesion molecule (Lutheran blood group)
BCL6: B-cell CLL/lymphoma 6
BIND: Biomolecular Interaction Network Database
BMP1: bone morphogenetic protein 1
bBTBD10: BTB (POZ) domain containing 10
C3: complement component 3
CALR or CRT: calreticulin
CCDC53: coiled-coil domain containing 53
CCSB-HI1: Center for cancer Systems Biology-Human Interactome
CD36: CD36 molecule (thrombospondin receptor)
CD44: CD44 molecule (Indian blood group)
CD47: CD47 molecule
CFH: complement factor H
cGMP: cyclic guanosine monophosphate
COL1A1: collagen, type I, alpha 1
COL2A1: collagen, type II, alpha 1
COL3A1: collagen, type III, alpha 1 (Ehlers–Danlos syndrome type IV, autosomal dominant)
COL5A1: collagen, type V, alpha 1
COL6A1: collagen, type VI, alpha 1
COL7A1: collagen, type VII, alpha 1 (epidermolysis bullosa, dystrophic, dominant and recessive)
CORO1A: coronin, actin binding protein, 1A
CSNK1A1: casein kinase 1, alpha 1, CSNK2A1, casein kinase 2, alpha 1 polypeptide
CSNK2A2: casein kinase 2, alpha prime polypeptide
CTGF: connective tissue growth factor
CTSG: cathepsin G
CUBN: cubilin (intrinsic factor-cobalamin receptor)
CYHR1: cysteine/histidinerich 1
DAG1: dystroglycan 1 (dystrophin-associated glycoprotein 1)
DCN: decorin
DHFR: dihydrofolate reductase
DIP: Database of Interacting Proteins
E3T3C1: Cterminal domain of TSP-1, containing the last type II repeat domain, the three type III repeat domains and the carboxy-terminal domain
ECM: extracellular matrix
EGF: epidermal growth factor
ELA2: elastase 2, neutrophil
ELN: elastin (supravalvular aortic stenosis, Williams–Beuren syndrome)
ERK 1/2: extracellular signal-regulated kinase
F2: coagulation factor II (thrombin)
FBLN2: fibulin 2
FCGR2A: Fc fragment of IgG, low affinity IIa, receptor (CD32)
FGA: fibrinogen alpha chain
FN1: fibronectin 1
GEMIN4: gem (nuclear organelle) associated protein 4
GFI1B: growth factor independent 1B transcription repressor, gp83 or Tcgp83, *T. cruzi* trypomastigote ligand or trypomastigote surface 83 kDa glycoprotein
HCASM: human coronary artery smooth muscle cell
HPRD: Human Protein Reference database
HRG: histidine-rich glycoprotein
HSPG2: heparan sulfate proteoglycan 2
INF-γ: Interferon gamma
IGFBP5: insulin-like growth factor binding protein 5
IL-12: interleukin 12
IPI: International Protein Index
ITGA1: integrin, alpha 1
ITGA2: integrin, alpha 2 (CD49B, alpha 2 subunit of VLA-2 receptor)
ITGA3: integrin, alpha 3 (antigen CD49C, alpha 3 subunit of VLA-3 receptor)
ITGA4: integrin, alpha 4 (antigen CD49D, alpha 4 subunit of VLA-4 receptor)
ITGA6: integrin, alpha 6 (alpha subunit of VLA-6 receptor)
ITGAV: integrin, alpha V (vitronectin receptor, alpha polypeptide, antigen CD51)
ITGB1: integrin, beta 1 (fibronectin receptor, beta polypeptide, antigen CD29 includes MDF2, MSK12)
ITGB3: integrin, beta 3 (platelet glycoprotein IIIa, antigen CD61)
ITGB4: integrin, beta 4
JAG1: jagged 1 (Alagille syndrome)
KNG1: kininogen 1
KEGG: Kyoto Encyclopedia of Genes and Genomes
LAMA1: laminin, alpha 1
LAMA2: laminin, alpha 2 (merosin, congenital muscular dystrophy)
LAMA3: laminin, alpha 3
LAMA5: laminin, alpha 5
LAMB1: laminin, beta 1
LAMB2: laminin, beta 2 (laminin S)
LAMB3: laminin, beta 3
LAMC1: laminin, gamma 1 (formerly LAMB2)
LAMC2: laminin, gamma 2
LAMC3: laminin, gamma 3
LGALS3: lectin, galactoside-binding, soluble, 3
LGALS3BP: lectin, galactoside-binding, soluble, 3 binding protein
LIM2: lens intrinsic membrane protein 2, 19kDa
LRP1: low density lipoprotein-related protein 1 (alpha-2-macroglobulin receptor)
LRP5: low density lipoprotein receptor-related protein 5
LTBP1: latent transforming growth factor beta binding protein 1
MATN2: matrilin 2
MEF: mouse embryo fibroblast
MEK: mitogen-activated protein kinase kinase
MEP1A: meprin A, alpha (PABA peptide hydrolase)
MiMI: Michigan Molecular Interactions
MMP-2: matrix metallopeptidase 2 (gelatinase A, 72kDa gelatinase, 72kDa type IV collagenase)
MMP-9: matrix metallopeptidase 9 (gelatinase B, 92 kDa gelatinase, 92 kDa type IV collagenase)
MUC7: mucin 7, secreted
MYOC: myocilin, trabecular meshwork inducible glucocorticoid response
NID1: nidogen-1
NID2: nidogen-2 (osteonidogen)
NO: nitric oxide
NT5E: 5′-nucleotidase, ecto (CD73)
NTSP: N-terminal domain of TSP-1
ONECUT1: one cut homeobox 1
PDE1A: phosphodiesterase 1A, calmodulin-dependent
PDEA: phosphodiesterase 6A, cGMP-specific, rod, alpha
PDGFB: platelet-derived growth factor beta polypeptide (simian sarcoma viral (v-sis) oncogene homolog)
PLAT: plasminogen activator, tissue
PLC: phospholipase C
PLG: plasminogen
PPI: protein–protein interaction
PTPRF: protein tyrosine phosphatase, receptor type, F
RAF: v-raf-1 murine leukemia viral oncogene
RAS: rat sarcoma viral oncogene homolog
RNAi: RNA interference
RPSA: ribosomal protein SA
SAA1: serum amyloid A1
SAA2: serum amyloid A2
SCARB2: scavenger receptor class B, member 2
SCD4: stearoyl-CoA desaturase 5
SDC1: syndecan 1
SDC3: syndecan 3
SIP1: survival of motor neuron protein interacting protein 1
SMAD2: SMAD family member 2
SNAPIN: SNAP-associated protein
SPARC: secreted protein, acidic, cysteine-rich (osteonectin)
SPP1: secreted phosphoprotein 1 (osteopontin, bone sialoprotein I, early T-lymphocyte activation 1)
SUFU: suppressor of fused homolog (Drosophila)
SV2A: synaptic vesicle glycoprotein 2A
SV2B: synaptic vesicle glycoprotein 2B
SV2C: synaptic vesicle glycoprotein 2C
Tc45: *T. cruzi* 45 kDA surface mucin
Tc85: *T. cruzi* 85 kDa surface glycoprotein
TcCRT: *T. cruzi* calreticulin
TFPI: tissue factor pathway inhibitor (lipoprotein-associated coagulation inhibitor)
TGF-α: transforming growth factor alpha
TGF-β: transforming growth factor beta
TGFB1: transforming growth factor, beta 1
TSP-1/THBS1: thrombospondin 1
TMPRSS6: transmembrane protease, serine 6
TNFα: tumor necrosis factor alpha
TNFAIP6: tumor necrosis factor alpha-induced protein 6
TNFRSF11B: tumor necrosis factor receptor superfamily, member 11b (osteoprotegerin)
TNFRSF1A: tumor necrosis factor receptor superfamily, member 1A
TRAP: thrombospondin-related anonymous protein
TP53: tumor protein p53
TSC2: tuberous sclerosis 2
ZNF512B: zinc finger protein 512B
ZNF8: zinc finger protein 8.
